# Association Between Prior Aspirin Use and Acute Respiratory Distress Syndrome Incidence in At-Risk Patients: A Systematic Review and Meta-Analysis

**DOI:** 10.3389/fphar.2020.00738

**Published:** 2020-05-19

**Authors:** Huoyan Liang, Xianfei Ding, Hongyi Li, Lifeng Li, Tongwen Sun

**Affiliations:** ^1^ General ICU, First Affiliated Hospital of Zhengzhou University, Henan Key Laboratory of Critical Care Medicine, Zhengzhou, China; ^2^ Cancer Centre, First Affiliated Hospital of Zhengzhou University, Zhengzhou, China

**Keywords:** aspirin, acute respiratory distress syndrome, at-risk, systematic review, meta-analysis

## Abstract

**Background:**

Recent studies have shown that prior antiplatelet drug use could ameliorate the risk and mortality of acute respiratory distress syndrome (ARDS). However, the connection between prior acetylsalicylic acid (aspirin) use and the risk of ARDS is unknown. Our primary objective was to perform a meta-analysis on the currently available studies to assess the association between aspirin use prior to ARDS onset and ARDS incidence in at-risk patients.

**Methods:**

Two investigators separately searched four research databases: MEDLINE, EMBASE, Cochrane Library, and Web of Science for relevant articles from the earliest available data through to July 14, 2019. In this paper, we performed a meta-analysis of the fixed effects model using the inverse variance-weighted average method to calculate the pooled odds ratios (ORs) and 95% confidence intervals (CIs). The primary outcome was risk of ARDS, and the secondary outcome was the hospital mortality of at-risk patients.

**Results:**

This article included seven studies altogether, enrolling 6,764 at-risk patients. Our meta-analysis revealed that, compared to non-aspirin use, prior aspirin use was linked with a significantly lower incidence of ARDS in at-risk patients (OR, 0.78; 95% CI, 0.64–0.96; *P* = 0.018) with low statistical heterogeneity (*I* ^2^ = 1.7%). Additionally, difference between prior aspirin use and non-aspirin use was not remarkable for hospital mortality in at-risk patients (OR, 0.88; 95% CI, 0.73–1.07; *P* = 0.204), and this analysis did not involve statistical heterogeneity (*I* ^2^ = 0%).

**Conclusions:**

This article indicates an association between prior aspirin use and a lower incidence of ARDS in at-risk patients, suggesting that aspirin use could potentially lower the risk of ARDS, and the investigation of such an effect is an interesting area for future clinical studies.

## Introduction

Acute respiratory distress syndrome (ARDS) is a clinical syndrome with high morbidity and mortality. ARDS is characterized by severely hypoxemic respiratory failure that requires mechanical ventilation and often induces organ failure. In addition, ARDS results in lower quality of life in patients after hospital discharge (Herridge et al., 2003; Rubenfeld et al., 2005; Herridge et al., 2011).

Mechanistically, ARDS is described as an inflammatory syndrome characterized by inflammatory injuries to the lungs mediated by immune cells, such as neutrophils and macrophages (Abraham, 2003; Frank et al., 2006; Perkins et al., 2007). The inflammatory response in the lungs is not under control and would lead to alveolar and capillary endothelial barrier injury, increasing its permeability to fluid. As the disease progresses, inflammatory factors, such as tumor necrosis factor alpha (TNF-α), interleukin (IL)-1, IL-6, and IL-12 increase, and inflammatory cells infiltrate the lungs. Moreover, an increase in proteinaceous fluid within the alveolar space leads to complete hypoxemia. Furthermore, widespread coagulation cascade activation causes microvascular thrombosis and fibroproliferation (Ware and Matthay, 2000). A previous study showed that albuterol could reduce the number of ventilator-free and organ failure-free days, yet it could not improve ARDS patient survival (Wu et al., 2015). At present, despite advancements in our understanding of ARDS pathogenesis, there remains a lack of effective therapy for ARDS (Boyle et al., 2013; Boyle et al., 2014).

Previous reports demonstrate that platelets play a substantial role in the onset of ALI or ARDS (Zarbock et al., 2007; Looney et al., 2009; Zarbock and Ley, 2009), as well as in the progression of these diseases (Fukunaga et al., 2005; El Kebir et al., 2009; Maderna and Godson, 2009). Experimental studies suggest that aspirin could ameliorate neutrophil activation and reduce inflammatory factors, such as TNF-α and IL-1 expression in lung macrophages, and it could also lower the levels of plasma thromboxane and reduce the degree of platelet sequestration in lung tissues (Chelucci et al., 1993; Yasuda et al., 2008). Unfortunately, this effect in clinical patients has been controversial. For instance, a prospective cohort study has shown that preadmission aspirin use was associated with a lower risk of ARDS (Chen et al., 2015). However, several other observational cohort studies (Kor et al., 2011; O'Neal et al., 2011; Tuinman et al., 2012; Boyle et al., 2015; Mazzeffi et al., 2015) and one RCT (Kor et al., 2016) have reported contrary results that, compared to non-aspirin use, prior aspirin use could not lower ARDS incidence. Numerous studies have demonstrated an effective therapeutic role of antiplatelet treatment in at-risk patients (Kor et al., 2011; Erlich et al., 2011; Chen et al., 2015; Boyle et al., 2015). A meta-analysis showed that aspirin use prior to the onset of sepsis could improve the mortality of septic patients (Mohananey et al., 2016); however, there is no relevant evidence that shows whether aspirin is associated with lower ARDS risk or hospital mortality in at-risk patients. Therefore, collecting the available data and assessing the collective effects of aspirin on ARDS incidence are necessary to resolve the current controversy.

## Materials and Methods

This meta-analysis was performed in accordance with the Preferred Reporting Items for Systematic Reviews and Meta-Analyses (**Additional File 1**) (Moher et al., 2009). The protocol of this meta-analysis has been registered in PROSPERO (http://www.crd.york.ac.uk/PROSPERO), and the registration number is **CRD42018103854**.

### Screening of Relevant Studies

**Figure 1:**
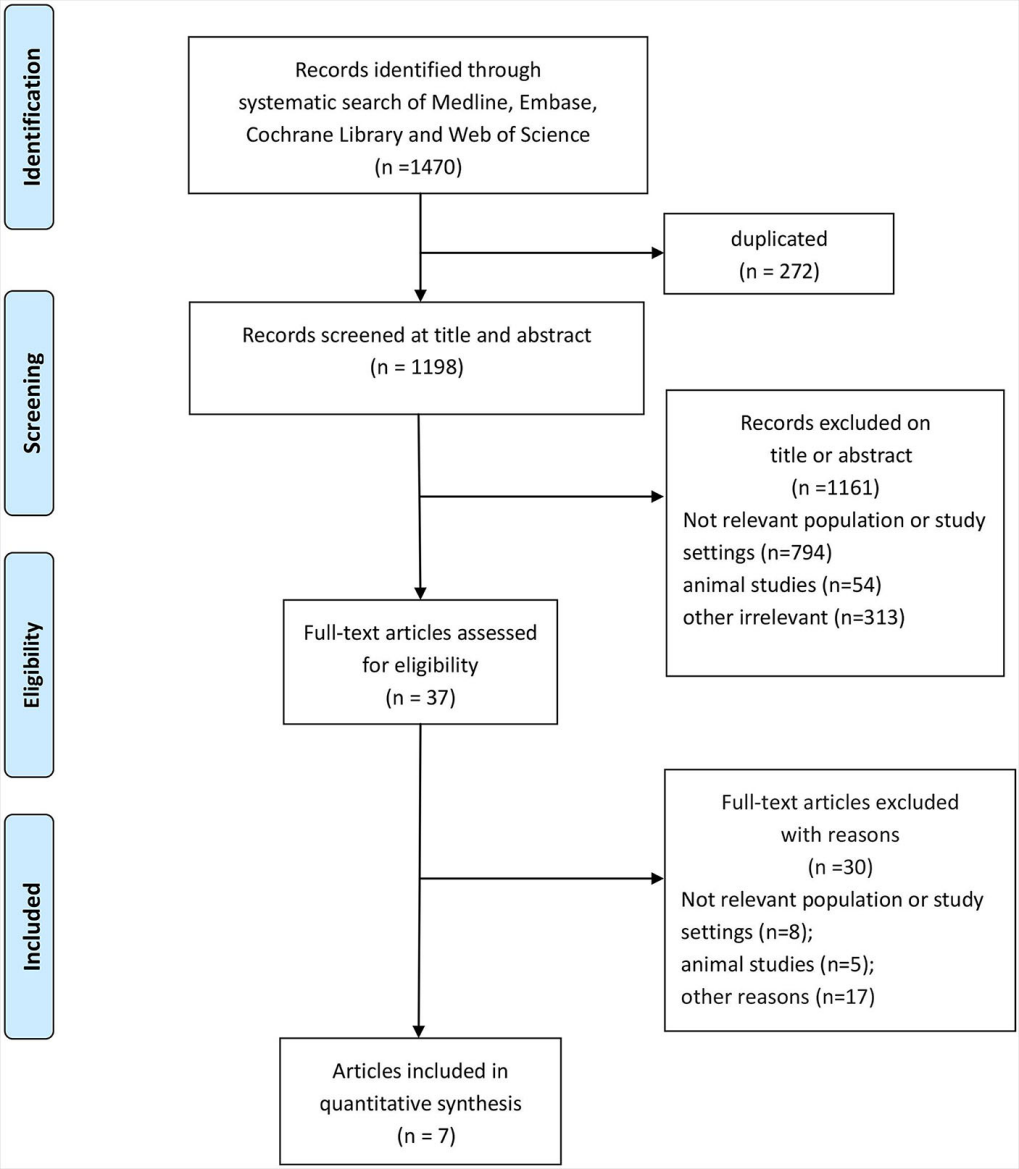
Flowchart of the study screening process.

A flowchart of the screening procedure for relevant studies is presented in **Figure 1**. We searched all the available data of MEDLINE, EMBASE, Cochrane Central Register of Controlled Trials (CENTRAL), and Web of Science until July 14, 2019. We also searched MeSH/Emtree and title/abstract terms for the combined key words, “acute lung injury”, “acute respiratory distress syndrome”, “critically ill patients”, “aspirin”, and “acetylsalicylic acid”. The search strategy for relevant studies are detailed in **Additional File 2**.

### Inclusion Criteria

This article includes the relevant studies to analyze the effects of aspirin on ARDS incidence in at-risk patients if: (1) the studies were observational cohort studies or randomized clinical trials (RCTs); (2) the study's participants were at-risk of ARDS or ARDS patients in the original articles; (3) the experimental arms of the studies were aspirin use prior to ARDS onset; (4) the control arms of the studies were at-risk ARDS patients who did not use aspirin or used placebo; (5) the studies measured the risk of ARDS or hospital mortality in at-risk patients that used aspirin and at-risk patients that did not use aspirin; and (6) the studies were published in the English language. The patients included in this meta-analysis were all adults. At-risk patients are defined as patients with the presence of at least one major risk factor for acute lung injury (ALI), including aspiration, pneumonia, sepsis, shock, pancreatitis, high-risk trauma, or high-risk surgery.

### Extraction of Data

First, two investigators (HoL and YY) extracted the data from the above four databases. In cases where disagreements occurred, the determination of whether the study would be included was discussed with the presence of a third reviewer (HuL). Then, the relevant data, including the first name of the first author in the original studies, the publication date, the represented country of the articles, the design type of the included studies, the number of experimental arms and control arms, the study period, the dose of aspirin used, the ARDS or acute lung injury definition, and the ARDS risk and hospital mortality were extracted from the original reports. Moreover, we evaluated the risk of ARDS as the primary outcome and hospital mortality as the secondary outcome.

### Risk of Bias Assessment

According to the principle of the Cochrane Collaboration (GS, 2011), all domains of bias in the RCT mainly included random sequence generation, allocation concealment, the blinding of participants, staff and outcomes assessors, incomplete outcome data, selective outcome reporting, and other biases. Additionally, we used the Newcastle-Ottawa Scale (NOS) to assess the risk of bias in the observational cohort studies (Sterne et al., 2016), and the items included the cohort selection, the comparability of the cohort design and analysis, as well as the adequacy of outcome measures. The total score was equal to or greater than seven points (high quality, **Additional File 3**).

### Statistical Analysis

The outcomes in this meta-analysis were the risk of ARDS and the hospital mortality in at-risk patients. In the meta-analysis, the heterogeneity included methodological heterogeneity, clinical heterogeneity, and statistical heterogeneity. Most of the meta-analysis contained statistical heterogeneity, which was evaluated by *I^2^* and *P* values. The degree of variability was due to the heterogeneity, not the sample error (Higgins and Thompson, 2002; Higgins et al., 2003), and when *I^2^* was less than 50%, between 51 and 75%, or equal to or greater than 76%, the heterogeneity was considered low, moderate, or high, respectively. Since the heterogeneity was low in ARDS risk and hospital mortality outcomes, we performed a meta-analysis of the fixed effects model using the inverse variance-weighted average method to calculate the pooled odds ratios (ORs) and 95% confidence intervals (CIs) for each patient outcome. To confirm the statistical significance, we performed a two-sided t-test, in which a *P*-value smaller than 0.05 was considered statistically significant. The software Stata 14.0 (College Station, Texas 77845 USA, Serial number: 401406267051) was used for all the statistical analyses in this meta-analysis.

## Results

### Screening of Relevant Studies

Based on the combined keywords, a total of 1,470 articles were found in our search of the four databases, 272 of which were duplicate studies. Among the remaining reports, 37 studies were considered potential articles based on their titles and abstracts. In the end, based on the inclusion criteria, seven studies (Kor et al., 2011; O'Neal et al., 2011; Tuinman et al., 2012; Chen et al., 2015; Boyle et al., 2015; Mazzeffi et al., 2015; Kor et al., 2016) were confirmed for meta-analysis after full text reading and judgment. The flowchart of the screening strategy for relevant studies is shown in **Figure 1**.

### Study Characteristics

A total of seven articles (Kor et al., 2011; O'Neal et al., 2011; Tuinman et al., 2012; Chen et al., 2015; Boyle et al., 2015; Mazzeffi et al., 2015; Kor et al., 2016) with 6,764 at-risk patients were included in this study. All patients were considered at risk of ARDS or diagnosed with ARDS. Additionally, the eligible participants in this study were the patients with ARDS/ALI, or with the presence of at least one major risk factor for acute lung injury, including aspiration, pneumonia, sepsis, shock, pancreatitis, high-risk trauma, or high-risk surgery. Moreover, the outcomes were the risk of ARDS/ALI and hospital mortality, and the data extracted from all eligible articles were the risk adjusted OR and 95% CI. In case the OR and its 95% CI were not reported, it was computed using related data reported in the original articles. Moreover, the first name of the first author in the original studies, the publication date, the represented country of the articles, the design type of the included studies, the number of the experimental arms and control arms, the study period, the dose and duration of aspirin use, and the ARDS or acute lung injury definition were also extracted. Further details of the study populations and interventions are shown in **Table 1**.

**Table 1:** Characteristics of the included studies.

Author (year)	Country	S			P		I		C	O	Quality score
		Study design	MC/SC	Study period	At-risk patients (participants)	ALI/ARDS definition	Dose and duration of aspirin use	Adjusted confounders	No. of arms (aspirin/non-aspirin)	Reported outcomes	
Boyle et al. (2015)	UK	PS	SC	Dec-10–07/2012	ARDS patients	The North American-European consensus	75–300 mg/daily	Age, APACHE II score, Coronary artery disease, PaO_2_ /FiO_2_ ratio, Vasopressor use	56/146	ICU mortality; duration of ICU stay; hospital mortality.	7
Chen et al. (2015)	US	PS	SC	23/01/2006–18/02/2012	Critically ill patients	Berlin definition	81 mg/d, 325 mg/d	Age, gender, race, sepsis and APACHE II score	287/862	Risk of ARDS; risk of sepsis.	8
Kor et al. (2011)	US	PS	MC	Mar-09–09/2009	Patients with at least one major risk factor for ALI	Standard American-European consensus	NA	Age, Sex (male), Admission Source, Diabetes Mellitus, Cirrhosis, Chronic Kidney Disease, Stage V, Congestive Heart Failure, Class IV, Chronic Obstructive Pulmonary Disease, Gastroesophageal Reflux Disease, Immunosuppression, ACE-I/ARB, Statin, Amiodarone	976/2879	Development of ARDS; ICU and hospital mortality; ICU and hospital length of stay.	7
Mazzeffi et al. (2015)	US	RS	SC	1/7/2008–30/06/2013	Patients who had AVRS during a 5-year period	Berlin definition	81 mg/d during the study period	Age, Cerebral vascular disease, Congestive heart failure, Diabetes mellitus, Dyslipidemia, Dialysis dependent, Male sex, Height, Hypertension, Infectious endocarditis, International normalized ratio, Left ventricular ejection fraction, Peripheral vascular disease, Weigh	181/194	Occurrence of ARDS; nadir PaO_2_ /FiO_2_ ratio	7
O'Neal et al. (2011)	US	PS	SC	23/01/2006–01/04/2008	Critically ill patients	The North American-European consensus	81 mg or 365 mg daily use	Prehospital statin use, Age, Gender, Current Tobacco Use, Race, APACHE II score	149/462	ICU mortality; duration of ICU stay; hospital mortality	7
Kor et al. (2016)	US	RCT	MC	02/07/2012–17/11/2014	Patients with LIPS ≥ 4	Berlin definition	325 mg loading dose followed by 81 mg/d for 7 d	NA	195/195	Development of ARDS; ventilator-free days to hospital 28 d; ICU and hospital lengths of stay; 28 d mortality.	7
Tuinman et al. (2012)	Netherlands	PS	SC	NA	Critically ill patients	2004 consensus definition	80 mg/d or 100 mg/d for 30 d	Amount of RBCs, FFP, PLTs and propensity score	109/109	Incidence of transfusion-related ALI	8

United Kingdom; US, United States; PS, prospective study; RS, retrospective study; RCT, randomized clinical trial; SC, single center; MC, multicenter; ICU, intensive care unit; ARDS, acute respiratory distress syndrome; ALI, acute lung injury; LIPS, lung injury prediction score; AVRS, aortic valve replacement surgery; NA, not applicable.

### Assessing the Risk of Bias

Six of the included articles (Kor et al., 2011; O'Neal et al., 2011; Tuinman et al., 2012; Chen et al., 2015; Boyle et al., 2015; Mazzeffi et al., 2015; Kor et al., 2016) were observational cohorts and one was an RCT (Kor et al., 2016), all of which had the risk of bias scores equal to or greater than seven points, suggesting a low risk of bias (**Table 1** and **Additional File 3**).

### Effects of Aspirin on Patient Outcomes

**Figure 2:**
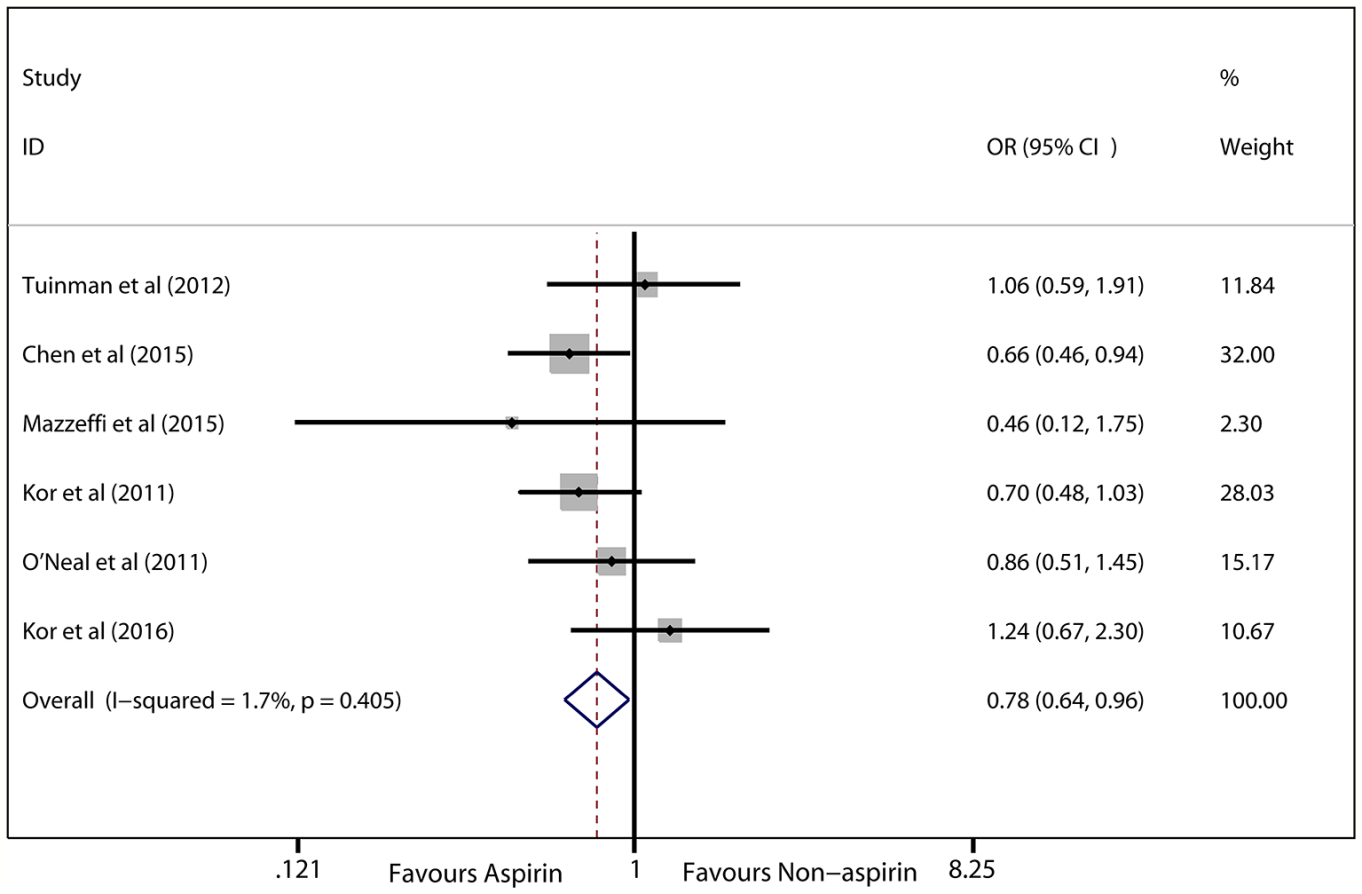
Forest plot showing the association between prior aspirin use and ARDS incidence in at-risk patients. The synthetic odds ratios (ORs) of the included studies indicate that prior aspirin use could decrease the incidence of ARDS in at-risk patients based on the fixed effects model.

**Figure 3:**
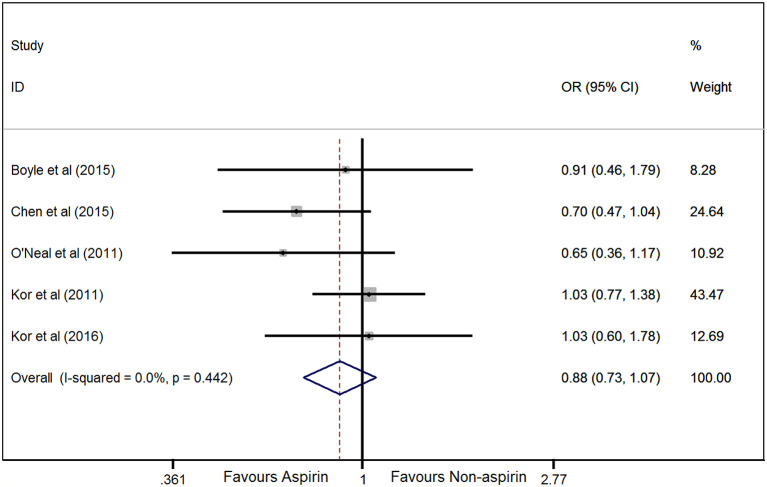
Forest plot showing the association between prior aspirin use and hospital mortality in at-risk patients. The pooled odds ratios (ORs) of the included studies indicate that prior aspirin use cannot decrease hospital mortality in at-risk patients, according to the fixed effects model.

In the seven eligible studies (Kor et al., 2011; O'Neal et al., 2011; Tuinman et al., 2012; Chen et al., 2015; Boyle et al., 2015; Mazzeffi et al., 2015; Kor et al., 2016), the risk of ARDS was chosen as the primary outcome (6,764 patients). Prior aspirin use was significantly associated with a decreased risk of ARDS in at-risk patients (OR, 0.78; 95% CI, 0.64–0.96; *P* = 0.018; *I* ^2^ = 1.7%; **Figure 2**). Hospital mortality was treated as the secondary outcome, and the difference between prior aspirin use and non-aspirin use for hospital mortality in at-risk patients was not significant (OR, 0.88; 95% CI, 0.73–1.07; *P* = 0.204; *I* ^2^ = 0%; **Figure 3**).

### Sensitivity Analysis

**Figure 4:**
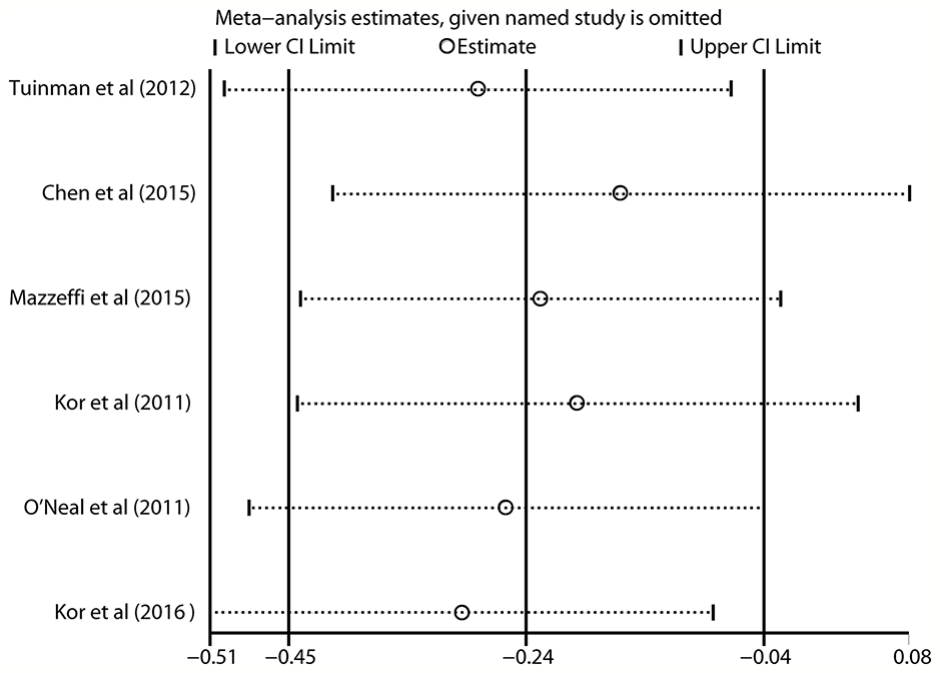
Sensitivity analysis indicating that the included studies were conclusive and reliable regarding the use of aspirin and the risk of ARDS in at-risk patients.

Since all of the included studies were observational studies or an RCT with a low risk of bias (**Table 1**), the sensitivity analysis was not conducted on the meta-analysis, according to the methodological criteria. Instead, the analysis was used to evaluate the effect of each study on the collective OR and 95% CI by omitting articles one at a time. Our data suggested that the results were robust and reliable (**Figure 4** and **Supplemental Figure 1**).

### Publication Bias

**Figure 5:**
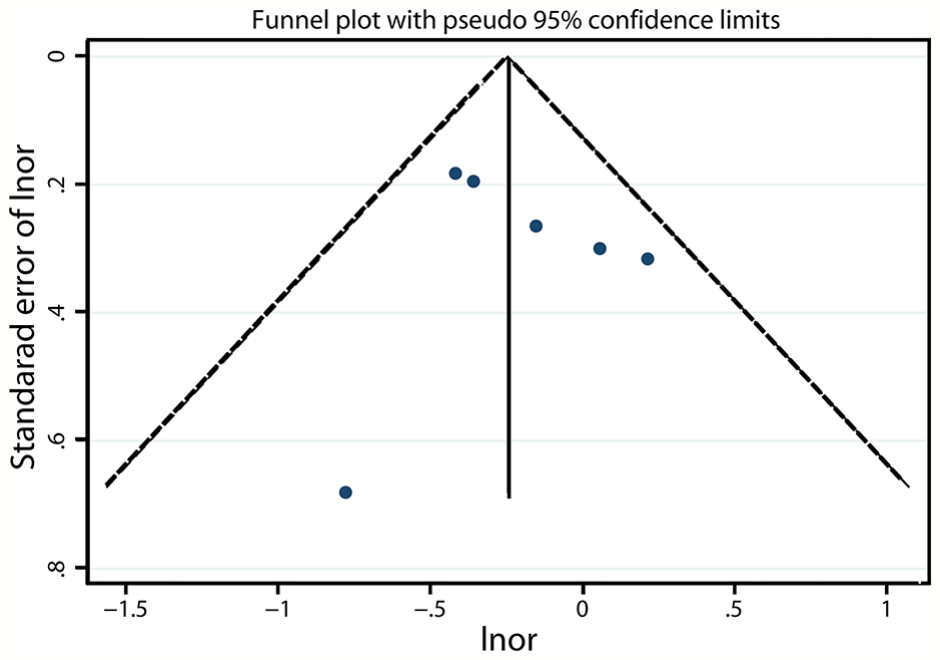
Funnel plot assessing the risk of ARDS after prior aspirin use in at-risk patients.

**Figure 6:**
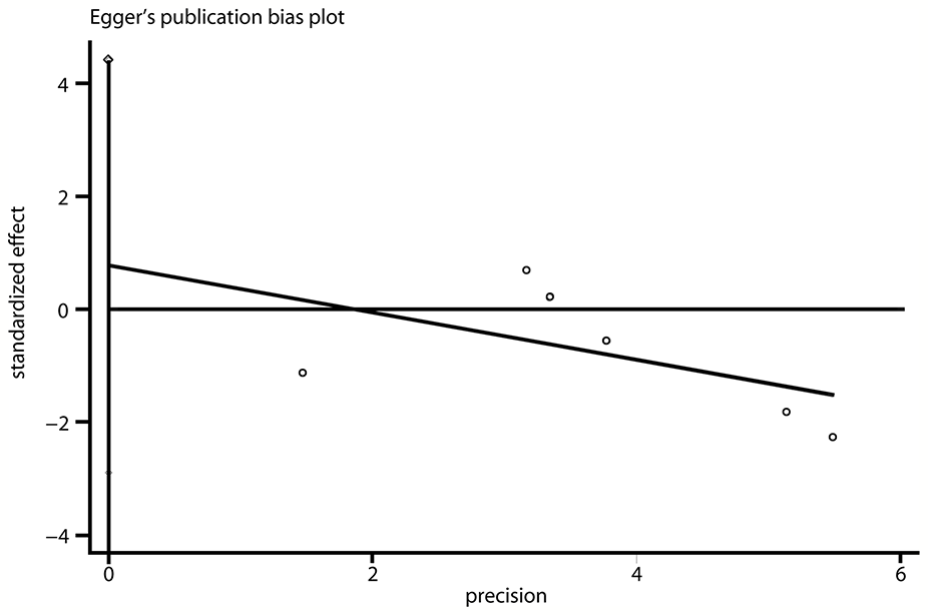
Egger regression line evaluating the publication bias of the included studies.

A funnel plot revealed that no asymmetry was found upon visual inspection (**Figure 5** and **Supplemental Figure 2**). The Egger linear regression test showed that there was no potential publication bias (*P* = 0.600 and 0.544; **Figure 6** and **Supplemental Figure 2 and 3**, respectively).

## Discussion

This article includes seven studies with 6,764 patients. Our meta-analysis demonstrates that prior aspirin use could improve the risk of ARDS incidence, but may not decrease hospital mortality in at-risk patients. To the best of our knowledge, this is the first time that systematic evidence was found for describing the effect of prior aspirin use on ARDS risk and hospital mortality in at-risk patients. However, further clinical studies are necessary to explore whether aspirin is efficacious in the hospital in at-risk patients previously untreated by aspirin.

Leeman et al. (1988) first reported that aspirin could decrease the intrapulmonary shunt in pig ALI model by decreasing the prostacyclin level or increasing leukotriene level. Subsequently, Zarbock et al. (2006) demonstrated that an important feature of ARDS is platelet neutrophil aggregation, and aspirin could reduce the platelet neutrophil aggregation and increase the gas exchange, thereby decreasing the mortality of ARDS animal model. However, the Gongalves de Moraes et al. (1996) showed that aspirin treatment could increase the neutrophil number in the bronchial alveolar fluid in the ALI mouse model. Interestingly, a systematic review (Panka et al., 2017) showed that aspirin could attenuate inflammation and pulmonary edema and improve the survival of preclinical ARDS models. These preclinical experiments provided evidence for the use of aspirin in future clinical studies. A randomized clinical trial (Kor et al., 2016) reported that aspirin did not reduce the incidence of ARDS, which do not support continuation to a larger clinical trial, which was inconsistent with the conclusion of our meta-analysis. Importantly, a subsequent prospective human trial (Hamid et al., 2017) indicated that aspirin can inhibit pulmonary neutrophilic inflammation, at both low and high doses, which was agreed with the potential effect of aspirin treatment for ARDS in our study, and the further clinical studies should be required and evaluate the aspirin for the prevention and treatment of ARDS.

Several mechanisms underlying the effect of aspirin on improving the risk of ARDS in at-risk patients have been elucidated. Numerous studies have shown that immune cells, especially macrophages and neutrophils, migrate to and activate in the lung endothelium, possibly inducing alveolar-capillary membrane injury (Kodama et al., 2007; Perl et al., 2008). Furthermore, studies indicate that inflammatory factors could activate platelets, change platelet granule secretion and platelet shape that regulate immune function and the inflammatory response, and subsequently promote the onset and progression of ARDS (Tabuchi and Kuebler, 2008; Zarbock and Ley, 2009; Yadav and Kor, 2015). Mechanistically, aspirin is an antiplatelet drug that may exert anti-inflammatory functions, such as decreasing nuclear factor κB and intracellular adhesion molecule-1 expression. Interestingly, aspirin can also reduce levels of the platelet, p-selectin (Grommes et al., 2012; Papadakos, 2013; Tuinman et al., 2013; Eickmeier et al., 2013). Moreover, studies also attest to the ability of aspirin to inhibit thromboxane A2 production by acetylating cyclooxygenase-1, thereby ameliorating aggregation of neutrophils within the lung (Looney et al., 2009; Grommes et al., 2012). Furthermore, a review reported that aspirin acetylates cyclooxygenase-1, which inhibits its activity and thereby restrains the expression of prostaglandin and leukotriene, resulting in the alleviation of the inflammatory response (Awtry and Loscalzo, 2000). Aspirin was also confirmed to protect endothelial cells from activated platelets and to subsequently reduce the number of inflammatory cells as well as the levels of inflammatory factors and adhesion molecules (Marik, 2001). Furthermore, several studies have revealed that aspirin could improve the pulmonary barrier and lung injury by promoting the resolution of lipoxins, leading to pivotal anti-inflammatory responses and pro-resolution bioactions (Claria and Serhan, 1995; Chiang et al., 2005; Jin et al., 2007). A growing body of evidence shows that aspirin functions to decrease the intrapulmonary shunt, protect from pulmonary hypertension, and ameliorate lung edema and inflammation, thereby decreasing the risk of ARDS (Sigurdsson et al., 1989; Leeman et al., 1992).

Our meta-analysis used a synthetic statistical analysis of the available data to provide systematic evidence to resolve the aforementioned controversy regarding the aspirin use in the clinic. A meta-analysis is considered to be statistically heterogeneous when the difference of the outcomes in each study is beyond what is expected. In this meta-analysis, the pooled effect of whether ARDS risk or hospital mortality showed a low degree of heterogeneity reflected that the included studies exhibited no differences.

This article is advantageous in a number of ways. First, the results of the original studies were adjusted for confounding factors, which means the pure effect of prior aspirin use on ARDS risk and hospital mortality was obtained. Second, the sample size from each study was relatively large to ensure the sample is representative to produce robust results. Third, one RCT was enrolled in this meta-analysis, demonstrating the high quality of the original articles and making our study more conclusive and reliable. Fourth, the risk of bias assessment indicated a low risk of bias in all the included studies. Fifth, a fixed-effect model with generic inverse variance was performed, and the adjusted ORs and 95% CIs were extracted, so that the collective outcome of the ORs for the effect of aspirin on ARDS risk and hospital mortality could be calculated. Finally, the sensitivity analysis revealed that the results of this study were conclusive and reliable.

This meta-analysis also has several limitations. We studied the association between prior aspirin use and ARDS risk or hospital mortality in at-risk patients; however, only one RCT investigated such an association, and how long one should take aspirin to prevent ARDS ideally has not been determined. Further clinical studies are necessary to explore whether aspirin is efficacious in the hospital in at-risk patients previously untreated by aspirin. Although at-risk patients may benefit from prior aspirin use, the effects, safety, and dose of aspirin administration, initiation, and continuation in patients who are at risk of ARDS still require exploration.

## Conclusions

This is the first article to elucidate the efficacy of prior aspirin use on ARDS risk and hospital mortality in at-risk patients. Our results indicate that, in at-risk patients, prior aspirin use is associated with a decreased risk of ARDS, but prior aspirin use and hospital mortality are not significantly associated. However, the results presented in this article should be further confirmed by clinical studies.

## Data Availability Statement

The related data supporting the conclusions of this article will be made available by the authors, without undue reservation, to any qualified researcher.

## Author Contributions

All authors contributed essentially to the work presented in this article. TS and HuL conceived of the study. HoL and XD contributed to the data interpretation. HuL contributed to the study protocol and wrote the article. XD contributed all the figures. LL polished the language. TS revised the article.

## Funding

The Scientific and Technological Innovation leaders in Central Plains (Grant No. 194200510017), Provincial Ministry Co-construction Project from Medical Scientific and Technological Research Program of Henan Province (Grant No. SBGJ2018020), and Natural Science Foundation of Henan Province (Grant No. 182300410369) supported this study.

## Conflict of Interest

The authors declare that the research was conducted in the absence of any commercial or financial relationships that could be construed as a potential conflict of interest.

## Supplementary Material

Supplemental Figure 1 Sensitivity analysis indicating that the included studies were conclusive and reliable regarding the use of aspirin and the hospital mortality of ARDS in at-risk patients.

Supplemental Figure 2 Funnel plot assessing the hospital mortality of ARDS after prior aspirin use in at-risk patients.

Supplemental Figure 3 Egger regression line evaluating the publication bias of the hospital mortality of ARDS after prior aspirin use in at-risk patients in the included studies.

## Abbreviations

ARDS, acute respiratory distress syndrome; OR, odds ratio; CI, confidence interval; TNF-α, tumor necrosis factor alpha; IL, interleukin; NOS, Newcastle-Ottawa Scale; CENTRAL, Cochrane Central Register of Controlled Trials; RCTs, randomized clinical trial; ALI, acute lung injury.
