# Corrigendum: Association Between Prior Aspirin Use and Acute Respiratory Distress Syndrome Incidence in At-Risk Patients: A Systematic Review and Meta-Analysis

**DOI:** 10.3389/fphar.2020.583449

**Published:** 2020-09-30

**Authors:** Huoyan Liang, Xianfei Ding, Hongyi Li, Lifeng Li, Tongwen Sun

**Affiliations:** ^1^ General ICU, First Affiliated Hospital of Zhengzhou University, Henan Key Laboratory of Critical Care Medicine, Zhengzhou, China; ^2^ Cancer Centre, First Affiliated Hospital of Zhengzhou University, Zhengzhou, China

**Keywords:** aspirin, acute respiratory distress syndrome, at-risk, systematic review, meta-analysis

In the original article, there was a mistake in **Table 1** as published. The studies from Chen et al. and O’Neal et al. were conducted in United States rather than China and UK**.** The corrected **Table 1** appears below.

The authors apologize for this error and state that this does not change the scientific conclusions of the article in any way. The original article has been updated.

**Table 1:** Characteristics of the included studies.

Author (year)	Country	S			P		I		C	O	Quality score
		Study design	MC/SC	Study period	At-risk patients (participants)	ALI/ARDS definition	Dose and duration of aspirin use	Adjusted confounders	No. of arms(aspirin/non-aspirin)	Reported outcomes	
Boyle et al. (2015)^[20]^	UK	PS	SC	12/2010 -07/2012	ARDS patients	North AmericaEuropean consensus	75-300 mg/daily	Age, APACHE II score, Coronary artery disease, PaO_2_/FiO_2_ ratio, Vasopressor use	56/146	ICU mortality; duration of ICU stay; hospital mortality.	7
Chen et al. (2015)^[19]^	US	PS	SC	23/01/2006 -18/02/2012	Critically ill patients	Berlin definition	81 mg/d, 325 mg/d	Age, gender, race, sepsis and APACHE II score	287/862	Risk of ARDS; risk of sepsis.	8
Kor et al. (2011)^[21]^	US	PS	MC	03/2009 -09/2009	Patients with at least one major risk factor for ALI	Standard American-European consensus	NA	Age, Sex (male), Admission Source, Diabetes Mellitus, Cirrhosis, Chronic Kidney Disease, Stage V, Congestive Heart Failure, Class IV, Chronic Obstructive Pulmonary Disease, Gastroesophageal Reflux Disease, Immunosuppression, ACE-I/ARB, Statin, Amiodarone	976/2879	Development of ARDS; ICU and hospital mortality; ICU and hospital length of stay.	7
Mazzeffi et al. (2015)^[22]^	US	RS	SC	01/07/2008 -30/06/2013	Patients who had AVRS during a 5-year period	Berlin definition	81 mg/d during the study period	Age, Cerebral vascular disease, Congestive heart failure, Diabetes mellitus, Dyslipidemia, Dialysis dependent, Male sex, Height, Hypertension, Infectious endocarditis, International normalized ratio, Left ventricular ejection fraction, Peripheral vascular disease, Weigh	181/194	Occurrence of ARDS; nadir PaO_2_/FiO_2_ ratio	7
O’Neal et al. (2011)^[23]^	US	PS	SC	23/01/2006-01/04/2008	Critically ill patients	The North American-European consensus	81 mg or 365 mg daily use	Prehospital statin use, Age, Gender, Current Tobacco Use, Race, APACHE II score	149/462	ICU mortality; duration of ICU stay; hospital mortality	7
Kor et al. (2016)^[25]^	US	RCT	MC	02/07/2012- 17/11/2014	Patients with LIPS ≥ 4	Berlin definition	325 mg loading dose followed by 81 mg/d for 7 d	NA	195/195	Development of ARDS; ventilator- free days to hospital 28 d; ICU and hospital lengths of stay; 28 d mortality.	7
Tuinman et al (2012)^[24]^	Netherlands	PS	SC	NA	Critically ill patients	2004 consensus definition	80 mg/d or 100 mg/d for 30 d	Amount of RBCs, FFP, PLTs and propensity score	109/109	Incidence of transfusion -related ALI	8

