# Supplementary figures and images for: Association Between Prior Aspirin Use and Acute Respiratory Distress Syndrome Incidence in At-Risk Patients: A Systematic Review and Meta-Analysis
Source: Front Pharmacol. 2020 May 19;11:738. doi: 10.3389/fphar.2020.00738 (PMC7248262; doi:10.3389/fphar.2020.00738)

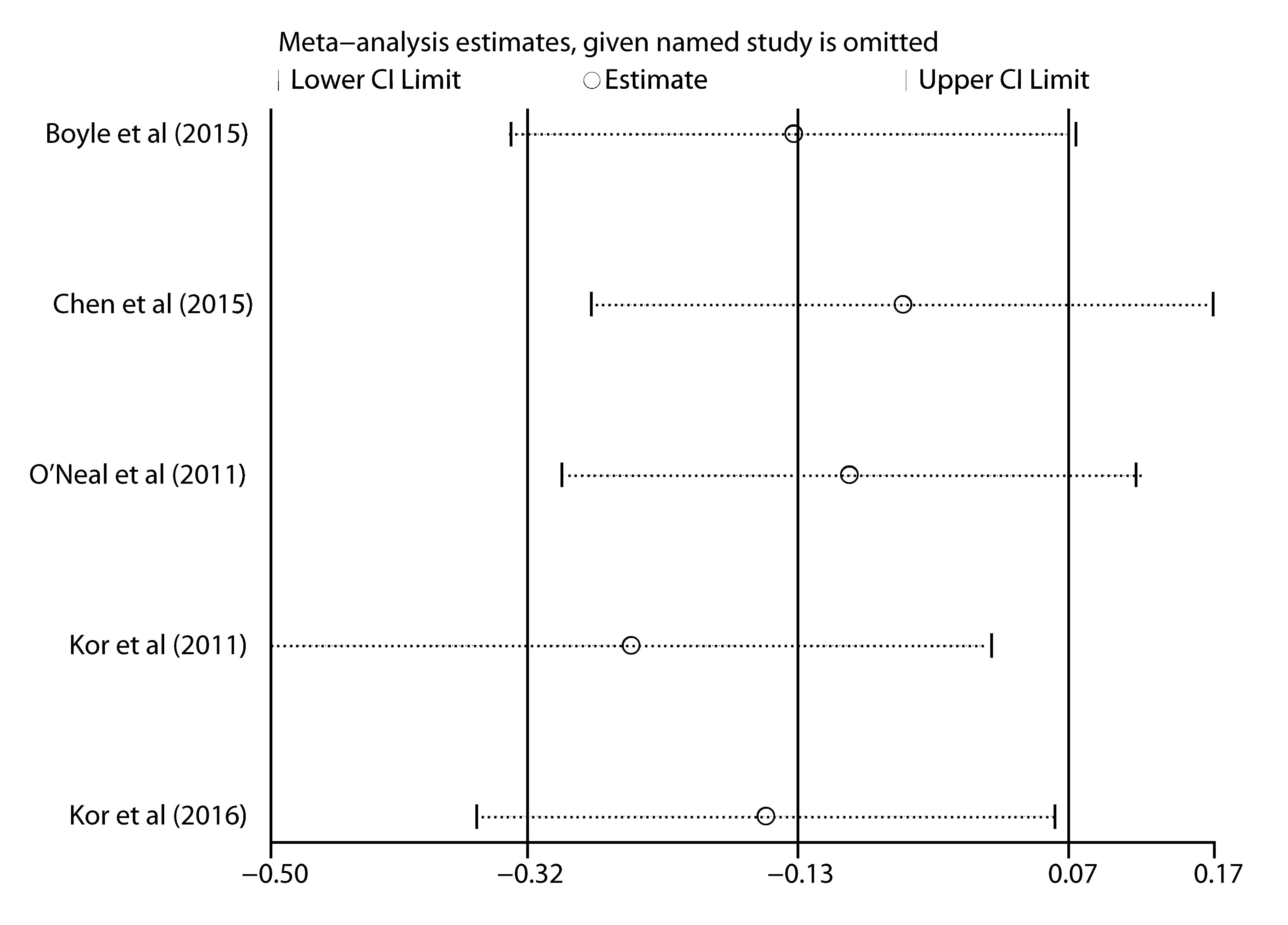

Supplement: Supplemental Figure 1 — Sensitivity analysis indicating that the included studies were conclusive and reliable regarding the use of aspirin and the hospital mortality of ARDS in at-risk patients. [file Image_1.tif]

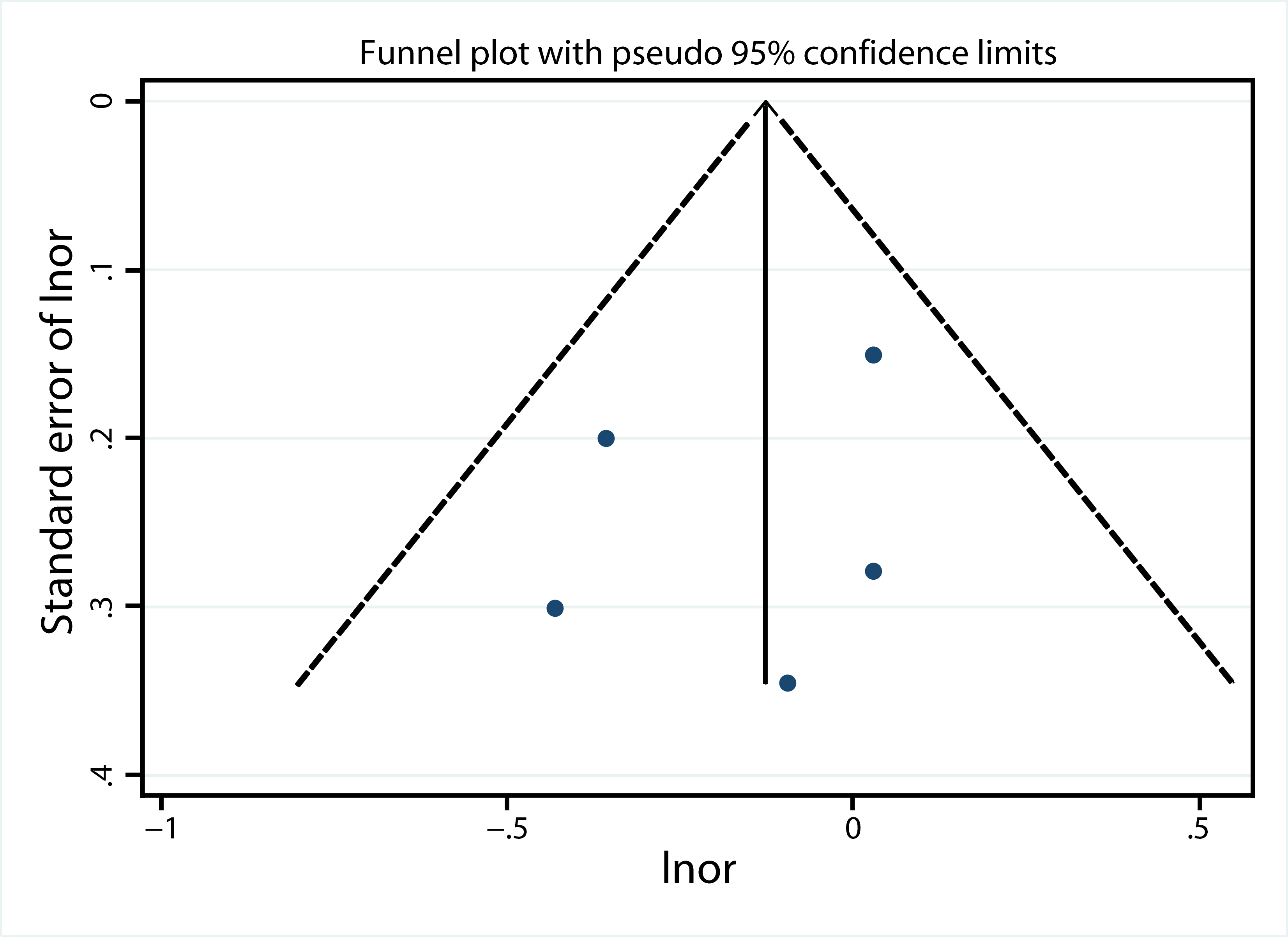

Supplement: Supplemental Figure 2 — Funnel plot assessing the hospital mortality of ARDS after prior aspirin use in at-risk patients. [file Image_2.tif]

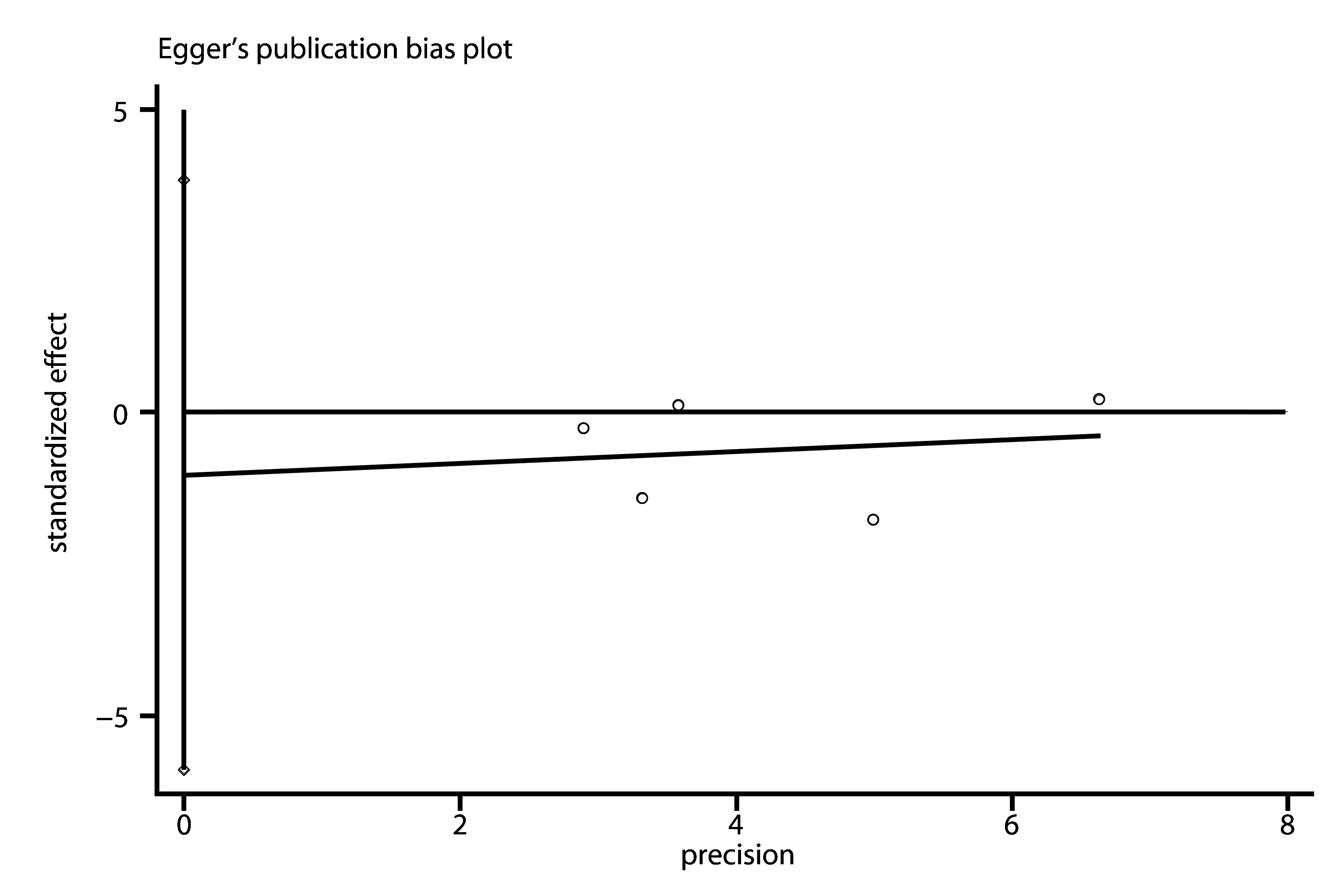

Supplement: Supplemental Figure 3 — Egger regression line evaluating the publication bias of the hospital mortality of ARDS after prior aspirin use in at-risk patients in the included studies. [file Image_3.tif]
