# Supplementary material for: Association Between Prior Aspirin Use and Acute Respiratory Distress Syndrome Incidence in At-Risk Patients: A Systematic Review and Meta-Analysis
Source: Front Pharmacol. 2020 May 19;11:738. doi: 10.3389/fphar.2020.00738 (PMC7248262; doi:10.3389/fphar.2020.00738)
Supplement: Supplementary file 5 [file Table_2.docx]

 **Additional file 2. The detailed search strategy**

| **Electronic databases** | **Search** | **Search strategy** | **Results** | **Links** |
| --- | --- | --- | --- | --- |
| **Pubmed** | #1 | Search ((((acute lung injury[MeSH Terms]) OR acute lung injury[Title/Abstract])) OR ((((critically ill patients[MeSH Terms]) OR critically ill patients[Title/Abstract])) OR ((Acute respiratory distress syndrome[Title/Abstract]) OR Acute respiratory distress syndrome[MeSH Terms])))) | 57781 | <https://www.ncbi.nlm.nih.gov/pubmed/?cmd=HistorySearch&querykey=4> |
|  | #2 | Search ((((((Aspirin[Title/Abstract]) OR Aspirin[MeSH Terms]) OR acetylsalicylic acid[Title/Abstract]) OR acetylsalicylic acid[MeSH Terms])) OR antiplatelet[Title/Abstract]) OR antiplatelet[MeSH Terms])))) | [84135](https://www.ncbi.nlm.nih.gov/pubmed/?cmd=HistorySearch&querykey=6) | <https://www.ncbi.nlm.nih.gov/pubmed/?cmd=HistorySearch&querykey=5> |
|  | #3 | Search((((((((Aspirin[Title/Abstract]) OR Aspirin[MeSH Terms]) OR acetylsalicylic acid[Title/Abstract]) OR acetylsalicylic acid[MeSH Terms])) OR antiplatelet[Title/Abstract]) OR antiplatelet[MeSH Terms])) AND (((((acute lung injury[MeSH Terms]) OR acute lung injury[Title/Abstract])) OR ((((critically ill patients[MeSH Terms]) OR critically ill patients[Title/Abstract])) OR ((Acute respiratory distress syndrome[Title/Abstract]) OR Acute respiratory distress syndrome[MeSH Terms])))) | [**192**](https://www.ncbi.nlm.nih.gov/pubmed/?cmd=HistorySearch&querykey=11) | <https://www.ncbi.nlm.nih.gov/pubmed/?cmd=HistorySearch&querykey=6> |
| **EMBASE** | #1 | 'adult respiratory distress syndrome'/de OR 'adult respiratory distress syndrome':ab,ti OR 'acute lung injury':ab,ti OR 'acute lung injury'/de OR 'critically ill patient':ab,ti OR 'critically ill patient'/de | 90,874 | <https://login.dcu.idm.oclc.org/login?qurl=https://www.embase.com%2f#advancedSearch/resultspage/history.1/page.1/25.items/orderby.date/source> |
|  | #2 | 'acetylsalicylic acid':ab,ti OR 'acetylsalicylic acid'/de OR aspirin:ab,ti OR 'aspirin'/de OR antiplatelet:ab,ti OR antiplatelet | 237,447 | <https://www-embase-com.dcu.idm.oclc.org/#advancedSearch/resultspage/history.2/page.1/25.items/orderby.date/source>. |
|  | #3 | #1 AND #2 | **746** | https://www-embase-com.dcu.idm.oclc.org/#advancedSearch/resultspage/history.3/page.1/25.items/orby.date/source |
| **Cochrane Library** | #1 | MeSH descriptor: [Respiratory Distress Syndrome, Adult] this term only | 1051 | <https://www-cochranelibrary-com.dcu.idm.oclc.org/advanced-search/search-manager> |
|  | #2 | MeSH descriptor:[Acute Lung Injury] this term only | 311 | <https://www-cochranelibrary-com.dcu.idm.oclc.org/advanced-search/search-manager> |
|  | #3 | MeSH descriptor:[Critical Illness] this term only | 1893 | <https://www-cochranelibrary-com.dcu.idm.oclc.org/advanced-search/search-manager> |
|  | #4 | MeSH descriptor: [Aspirin] this term only | 5431 | <https://www-cochranelibrary-com.dcu.idm.oclc.org/advanced-search/search-manager> |
|  | #5 | MeSH descriptor:[Platelet Aggregation Inhibitors] this term only | 3653 | <https://www-cochranelibrary-com.dcu.idm.oclc.org/advanced-search/search-manager> |
|  | #6 | (antiplatelet therapy):ti,ab,kw | 4618 | <https://www-cochranelibrary-com.dcu.idm.oclc.org/advanced-search/search-manager> |
|  | #7  #8  #9  #10  #11  #12  #13 | ("aspiring"):ti,ab,kw  ("critically ill"):ti,ab,kw  ("acute lung injury"):ti,ab,kw  ("acute respiratory distress syndrome"):ti,ab,kw  #1 or #2 or #3 or #8 or #9or #10  #4 or #5 or #6 or #7  #11 and #12 | 26  6100  1004  1321  8674  10784  **31** | <https://www-cochranelibrary-com.dcu.idm.oclc.org/advanced-search/search-manager>  <https://www-cochranelibrary-com.dcu.idm.oclc.org/advanced-search/search-manager>  <https://www-cochranelibrary-com.dcu.idm.oclc.org/advanced-search/search-manager>  <https://login.dcu.idm.oclc.org/login?qurl=https://www.cochranelibrary.com%2fadvanced-search%2fsearch-manager>  <https://www.cochranelibrary.com/advanced-search/search-manager>  <https://www.cochranelibrary.com/advanced-search/search-manager>  <https://www.cochranelibrary.com/advanced-search/search-manager> |
| **Web of Science** | #1 | TS= (antiplatelet* OR aspirin* OR acetylsalicylic acid) AND TS=( Acute respiratory distress syndrome* OR Acute lung injury* OR critically ill patients) | **521** | http://apps.webofknowledge.com/summary.do?product=UA&doc=1&qid=1&SID=7CaibGahLzBFwJamqan&search_mode=AdvancedSearch&update_back2search_link_param=yes |
