# Supplementary material for: Association Between Prior Aspirin Use and Acute Respiratory Distress Syndrome Incidence in At-Risk Patients: A Systematic Review and Meta-Analysis
Source: Front Pharmacol. 2020 May 19;11:738. doi: 10.3389/fphar.2020.00738 (PMC7248262; doi:10.3389/fphar.2020.00738)
Supplement: Supplementary file 6 [file Table_3.docx]

**The Newcastle-Ottawa Scale (NOS) for assessing the quality of cohort studies.**

| \| **Author** \| \| --- \| | **Selection** | | | **Comparability** | | | **Assessment of outcome** | | | **Total quality score** |
| --- | --- | --- | --- | --- | --- | --- | --- | --- | --- | --- | --- |
|  | **Representativeness of exposure arm (s)** | **Selection**  **of the comparative arm (s)** | **Origin of exposure source** | **Demonstration that outcome of interest was not present at start of study** | **Studies controlling the most important factors** | **Studies controlling the other main factors** | **Assessment of outcome with independency** | **Adequacy of follow-up length (to assess outcome)** | **Lost to follow-up acceptable (less than 10% and reported)** |  |
| Boyle et al (2015)^[17]^ | * | - | * | * | * | * | * | * |  | 7 |
| Chen et al (2015)^[18]^ | * | * | * | * | * | * | * | * |  | 8 |
| Kor et al (2011)^[20]^ | * | * | - | * | * | * | * | * |  | 7 |
| Mazzeffi et al (2015)^[28]^ | * | * | * | * | * | * | - | * |  | 7 |

**The Newcastle-Ottawa Scale (NOS) for assessing the quality of cohort studies.**

| **Author** | **Selection** | | | **Comparability** | | | **Assessment of outcome** | | | **Total quality score** |
| --- | --- | --- | --- | --- | --- | --- | --- | --- | --- | --- |
|  | **Representativeness of exposure arm (s)** | **Selection**  **of the comparative arm (s)** | **Origin of exposure source** | **Demonstration that outcome of interest was not present at start of study** | **Studies controlling the most important factors** | **Studies controlling the other main factors** | **Assessment of outcome with independency** | **Adequacy of follow-up length (to assess outcome)** | **Lost to follow-up acceptable (less than 10% and reported)** |  |
| O’Neal et al (2011)^[29]^ | * | * | - | * | * | * | * | * |  | 7 |
| Tuinman et al (2012)^[30]^ | * | * | * | * | * | * | * | * |  | 8 |

**RCT risk of bias recommended by the Cochrane Collaboration.**

| Author  (year) | random sequence generation |  | allocation concealment |  | blinding of  participants and staff |  | blinding of outcome assessors |  | incomplete  outcome data |  | selective outcome reporting |  | other biases |
| --- | --- | --- | --- | --- | --- | --- | --- | --- | --- | --- | --- | --- | --- |
| Kor et al (2016)^[27]^ |  |  |  |  |  |  |  |  |  |  |  |  |  |

Low risk bias

unclear

high risk bias
